# Circulating exosomal miR-550a-5p/miR-665 identify coronary microvascular dysfunction and drive endothelial–myocyte crosstalk in type 2 diabetes

**DOI:** 10.3389/fcell.2026.1744708

**Published:** 2026-01-29

**Authors:** Xiudong Ding, Guangliang Bai, Xin Liu, Yueming Dong, Yinghui Chai, Bing Han, Xianghong Meng, Hong Lei

**Affiliations:** 1 Department of Clinical Laboratory, The 8th Medical Center of PLA General Hospital, Beijing, China; 2 Department of Clinical Laboratory, No 984 Hospital of PLA, Beijing, China; 3 Department of Blood Transfusion, The 8th Medical Center of PLA General Hospital, Beijing, China

**Keywords:** coronary microvascular dysfunction, exosomes, hippo–YAP signaling, miR-550a-5p, miR-665, type 2 diabetes

## Abstract

**Objective:**

This study aimed to identify circulating exosomal microRNAs for early detection of coronary microvascular dysfunction (MD) and stratification of myocardial injury risk in type 2 diabetes, and to test whether miR-550a-5p derived from endothelial cell models mechanistically links endothelial stress to cardiomyocyte vulnerability via Hippo–eNOS signaling and vesicular transfer.

**Method:**

We combined Gene Expression Omnibus (GEO)-guided discovery and compendium prioritization with clinical and mechanistic validation. Plasma exosomal miR-550a-5p/miR-665 were quantified in a well-phenotyped Type 2 Diabetes Mellitus (T2DM) cohort and related to MD and myocardial injury. *In vitro*, miR-550a-5p was modulated in human cardiac microvascular endothelial cells under high glucose to profile Hippo–YAP/eNOS signaling, and its exosomal transfer effects on human cardiomyocytes were evaluated, with Hippo inhibition applied as a rescue strategy.

**Result:**

Exosomal miR-550a-5p and miR-665 were elevated in MD and further increased with myocardial injury, showing useful diagnostic performance and independent associations. miR-550a-5p inhibition restored endothelial viability, migration, tube formation and p-eNOS/eNOS, reduced MST1/LATS1 activation, and increased Yes-associated protein (YAP) activity. Endothelial exosomes delivered miR-550a-5p to cardiomyocytes, aggravating apoptosis and oxidative stress; donor-cell miR-550a-5p suppression and Hippo inhibition mitigated these effects.

**Conclusion:**

Exosomal miR-550a-5p, together with miR-665, functions as a dual-utility signal, serving as an accessible biomarker for coronary MD and as a mechanistic mediator in endothelial-to-cardiomyocyte exosomal signaling. These findings support earlier detection, risk stratification, and mechanism-guided intervention in T2DM. Future priorities include external validation, longitudinal kinetic assessment, and target-network mapping.

## 1 Introduction

Type 2 diabetes mellitus (T2DM) is a major driver of cardiovascular morbidity, and microvascular pathology is now recognized as a central pathophysiological component along the continuum from metabolic stress to organ dysfunction (Li et al., 2025; Liang et al., 2024; Meng et al., 2022; Barrea et al., 2025). In the heart, microvascular dysfunction (MD) compromises capillary density and endothelial signaling, limiting perfusion and adaptive reserve, thereby predisposing to cardiomyocyte injury, adverse remodeling, and ultimately diastolic dysfunction and heart failure with preserved ejection fraction (HFpEF) (Guan et al., 2019; Kaze et al., 2024; Yeo et al., 2022). Currently, in routine care, risk stratification still relies on systemic metabolic indices (e.g., HbA1c), nonspecific inflammatory surrogates, or downstream echocardiographic changes, all of which are suboptimal for early detection (Lyssenko and Vaag, 2023; Song et al., 2023). This gap motivates the pursuit of non-invasive circulating biomarkers that report both the presence of MD and the propensity for myocardial injury, ideally capturing real-time vascular–cardiac crosstalk under hyperglycemia (Veitch et al., 2022; Davoodian et al., 2025; Ma et al., 2022).

Extracellular vesicles (EVs)—particularly exosomes—offer a biologically grounded conduit for such biomarkers (Fluitt et al., 2022; Xue et al., 2024; Zhou et al., 2025). Exosomes are nano-sized lipid bilayer vesicles (≈30–150 nm) released by most cell types, carrying proteins, lipids, and nucleic acids (including microRNAs, miRNAs) (Prattichizzo et al., 2021; La Marca and Fierabracci, 2017). Because their cargos are membrane-protected and dynamically regulated by cellular stressors (e.g., oxidative stress and nutrient overload), exosomes reflect tissue states and can reprogram recipient cells both locally and systemically (Eissa et al., 2016; Chen et al., 2021). These properties underlie two complementary applications: (i) diagnostics, where circulating exosomal miRNAs serve as robust liquid-biopsy readouts of subclinical vascular injury; and (ii) therapeutics, where modifying exosome cargo can enhance endothelial repair and angiogenesis in diabetic settings (Huang et al., 2021). Together, this positions circulating exosomal miRNAs as analytically attractive and clinically accessible indicators for diabetes-related MD and its myocardial consequences (Veitch et al., 2022; Fan et al., 2025; Mori et al., 2014; Austin et al., 2019).

Recent experimental evidence substantiates the measurability and relevance of circulating EV miRNA signatures across the diabetic cardiovascular continuum (Li et al., 2022; Mo et al., 2023). In preclinical T2DM models, circulating EV-encapsulated miR-30d-5p/miR-30e-5p increased in step with endothelial oxidative stress, DNA damage/senescence, and coronary microvascular rarefaction (Lin et al., 2013). Notably, endothelial cells in the left ventricle are a likely source, and functional studies link miR-30 to derangements in fatty-acid metabolism, Reactive Oxygen Species (ROS) generation, and reduced eNOS, implicating miR-30 family members as early biomarkers and effectors of microvascular dysfunction relevant to HFpEF pathogenesis (Windmueller and Morrisey, 2015; Tian et al., 2015). These data provide a biological and analytical blueprint for using EV miRNAs to detect subclinical coronary MD (Veitch et al., 2022; Sharma et al., 2022).

Parallel work highlights how vascular miRNA programs are modifiable, with direct consequences for microvascular integrity and cardiac performance (Ji et al., 2023). In a comprehensive study of diabetic db/db mice, timely exercise (initiated prior to established dysfunction) preserved coronary structure and flow, reduced apoptosis and fibrosis, and normalized a set of cardiovascular-enriched miRNAs; mechanistic perturbation of miR-126 demonstrated that restoration of this endothelial miRNA was necessary for the exercise-mediated protection of the microcirculation (Lew et al., 2020). These findings support two key translational points: first, that miRNA-encoded information is central to microvascular–myocardial coupling in diabetes; and second, that trajectory-shaping interventions can be guided—or even monitored—by miRNA changes, strengthening the rationale to develop miRNA-based diagnostics upstream of overt dysfunction (Lew et al., 2020; Hu et al., 2018).

Beyond biomarker associations, endothelial–cardiomyocyte crosstalk via exosomes has been causally linked to diabetic cardiomyopathy (Xue et al., 2024). In a landmark endothelial-to-cardiomyocyte transfer study, cardiac microvascular endothelial cell (CMEC)-derived exosomes delivered the Hippo kinase Mst1 to cardiomyocytes, where increased Mst1 protein (without mRNA change) suppressed autophagy, promoted apoptosis, and impaired glucose handling by disrupting GLUT4 membrane translocation (Veitch et al., 2022). Endothelial Mst1 overexpression *in vivo* deteriorated cardiac function and aggravated insulin resistance under streptozotocin-induced diabetes, demonstrating that microvascular exosomal cargo can propagate maladaptive signals to cardiomyocytes under hyperglycemia. This endothelial-initiated, exosome-mediated mechanism links microvascular injury to cardiomyocyte death pathways and metabolic inflexibility, providing a conceptual bridge from biomarker to mechanism (Fan et al., 2025; Hu et al., 2018).

Importantly, the translational value of exosomal miRNAs as liquid-biopsy biomarkers has been demonstrated across diabetic organ systems, illustrating methodological feasibility and cross-species conservation (Lyssenko and Vaag, 2023; Lew et al., 2020; Spinetti et al., 2013). A recent pipeline integrating long-term diabetic rat models with human plasma profiling identified circulating exosomal miR-16–5p and let-7e-5p as fibrosis-associated candidates in diabetic cystopathy (Xue et al., 2024). The study combined differential expression, target and TF prediction, PPI network analysis, and regulatory network construction, and validated candidates by qPCR in plasma exosomes—an approach that can be ported to diabetes-related microvascular complications in other organs, including the heart (Fan et al., 2025). This work underscores that systemic diabetic stress imprints a reproducible miRNA signature on circulating exosomes that tracks fibrotic remodeling—a common end-organ pathway of microvascular injury.

Therapeutically, engineering or conditioning donor cells can reshape exosomal cargo to enhance vascular repair under diabetic stress. Hypoxia-primed urine-derived stem-cell exosomes, enriched for miR-486-5p, promoted endothelial proliferation, migration, and tube formation, and—when delivered via a bioactive hydrogel—accelerated *in vivo* vascular regeneration and wound closure in diabetic rats by targeting SERPINE1 (Fan et al., 2025). Although studied in cutaneous healing, the mechanistic logic—exosomal miRNA reprogramming of endothelial behavior—maps directly onto the vascular pathology of the diabetic heart, reinforcing that exosomal miRNAs are not only biomarkers but also effectors of microvascular remodeling.

Despite this progress, critical gaps remain. First, there is a scarcity of circulating exosomal miRNAs that simultaneously deliver diagnostic utility for MD and risk signals for myocardial injury within a coherent clinical framework in T2DM. Second, for specific and underexplored candidates (e.g., miR-550a-5p, miR-665), integrated evidence spanning bioinformatic discovery, clinical validation, and mechanistic causality is limited. Third, it remains to be determined whether particular exosomal miRNAs act through Hippo–YAP and eNOS axes within the endothelium and are transferred to cardiomyocytes to aggravate apoptosis, oxidative stress, and loss of viability. Addressing these questions would help establish a biomarker–mechanism continuum, enabling earlier detection and more precise risk stratification. It may also point to actionable pathways for intervention.

Accordingly, in the present study we integrated GEO-based discovery with clinical and mechanistic validation to identify and test circulating exosomal miRNAs linked to diabetes-related MD. We first intersected differentially expressed circulating exosomal miRNAs with disease-focused compendia and mapped targetomes and transcriptional regulators to prioritize candidates. Next, we verified their diagnostic performance for MD and quantified their associations with myocardial injury in a well-phenotyped T2DM cohort. Finally, using HCMEC–AC16 models, we delineated endothelial–cardiomyocyte exosomal transfer, interrogated Hippo pathway involvement, and assessed downstream effects on apoptosis, oxidative stress, and viability. By bridging biomarker discovery with mechanism, our work advances a pathway toward non-invasive detection of MD and targeted modulation of exosomal miRNA signaling in diabetic heart disease.

## 2 Materials and methods

### 2.1 Bioinformatic analysis

Circulating exosomal miRNA expression profiles were retrieved from the Gene Expression Omnibus (GEO) under accession GSE262614, comprising five diabetic cases and five healthy controls. Raw matrices and associated sample annotations were downloaded from GEO and imported into R (v4.x). When necessary, probe identifiers were mapped to mature human miRNA names according to the corresponding platform annotation; duplicated miRNAs were summarized by median expression. To stabilize variance, expression values were log2-transformed (adding a small offset where required by the platform) and samples were screened for outliers by principal component analysis before downstream modeling. Given the limited sample size of the public GEO dataset, this analysis was designed as an exploratory, hypothesis-generating step to nominate candidate miRNAs for downstream clinical validation and mechanistic investigation.

Differential expression between diabetic and control groups was assessed with the limma framework. A design matrix encoding group status was fitted to each miRNA with ordinary least squares, and empirical Bayes variance moderation (eBayes) was applied. Multiple testing was controlled by the Benjamini–Hochberg procedure; miRNAs meeting |log2 fold-change| >0.585 (≈1.5-fold) and FDR <0.05 were considered significant. The full distribution of effects and adjusted P values was visualized by a volcano plot, and unsupervised patterns among significant miRNAs were displayed by a hierarchical clustering heatmap (Euclidean distance, complete linkage), with samples annotated by group.

To focus the search on clinically relevant signals, a compendium of disease-associated miRNAs was curated from HMDD and miRNet, restricting entries to terms related to diabetes and microvascular/endothelial dysfunction. The intersection between this compendium and the GEO-derived differentially expressed miRNAs defined the candidate set for validation and mechanistic analyses.

Putative mRNA targets of candidate miRNAs were predicted independently using TargetScan (human, conserved sites) and miRDB; only interactions supported by both resources were retained to reduce false positives. The resulting target lists were harmonized to official HGNC symbols and converted to Entrez IDs with org.Hs.e.g.db, removing unmapped entries. Functional annotation was performed using clusterProfiler: GO enrichment was run across Biological Process, Cellular Component, and Molecular Function ontologies, and KEGG pathway enrichment was conducted in parallel. Unless otherwise stated, enrichment analyses used the set of all predicted targets as background, a two-sided hypergeometric test, and significance thresholds of adjusted *P* < 0.05 and q < 0.05. Bubble plots summarize representative GO terms and KEGG pathways.

To explore cooperative regulation at the protein level, predicted targets were submitted to STRING (*Homo sapiens*) to construct a protein–protein interaction (PPI) network. Edges were filtered at a combined score ≥0.7 (high confidence). Network topology was analyzed in Cytoscape with cytoHubba, and maximal clique centrality (MCC) was used to rank hub genes. In parallel, FunRich was used to predict transcription factors (TFs) likely to regulate the target set. An integrated miRNA–gene–TF regulatory network was then assembled in Cytoscape to depict candidate miRNAs, their top targets (including hubs), and enriched TFs in a single graph.

All plots (volcano, heatmap, enrichment bubbles, and network summaries) were generated in R using ggplot2/pheatmap/clusterProfiler and Cytoscape. Unless specified, analyses used default parameters; all tests were two-sided with FDR control as above.

### 2.2 Clinical specimens and data collection

This study enrolled adults with T2DM who received care. Inclusion criteria were: diagnosis of T2DM according to WHO (1999) or ADA (2023) criteria; disease duration ≥1 year; age 35–75 years; absence of acute diabetic complications within the preceding 3 months; availability of adequate peripheral venous blood for exosome isolation. Exclusion criteria comprised: known coronary artery disease, prior myocardial infarction, heart failure, or structural heart disease; clinically significant arrhythmia or uncontrolled hypertension; non-diabetic microvascular diseases (e.g., non-diabetic nephropathy or retinopathy); active malignancy, active infection, or autoimmune disease; and pregnancy or lactation.

After an overnight fast, peripheral venous blood was collected into EDTA tubes and processed within 2 h. Platelet-poor plasma was prepared by sequential centrifugation at 2,000 × g for 15 min and 10,000 × g for 30 min at 4 °C, with supernatants reserved for exosome isolation. Patients were stratified by the presence of microvascular dysfunction into MD and NMD groups according to institutional clinical protocols; the MD group was further subdivided into MD-MI and MD-NMI based on adjudicated myocardial injury recorded in the medical chart. Demographic and clinical variables were abstracted from electronic records, including age, sex, body mass index (BMI), hypertension, dyslipidemia, diabetes duration, HbA1c, and cardiac biomarkers (troponin T [TnT], troponin I [TnI], N-terminal pro-B-type natriuretic peptide [NT-proBNP], and creatine kinase-MB [CK-MB]). All procedures conformed to the Declaration of Helsinki and were approved by the Medical Ethics Committee of the Eighth Medical Center of the Chinese PLA General Hospital (Approval No. 3092024042250931).

### 2.3 Exosome isolation and characterization (concise)

Exosomes were isolated from platelet‐poor plasma by differential ultracentrifugation. Briefly, clarified plasma was passed through a 0.22-µm filter and ultracentrifuged at 100,000 × g for 70 min at 4 °C. Pellets were washed once in ice-cold PBS and re-spun at 100,000 × g for 70 min, then resuspended in PBS, quantified by BCA, and stored at −80 °C until analysis.

For cell-derived vesicles, HCMECs were cultured in medium containing exosome-depleted FBS (prepared by overnight spin at 100,000 × g) under normal- or high-glucose conditions. Conditioned media were cleared by sequential centrifugation (300 × g, 2,000 × g, 10,000–18,000 × g), filtered (0.22 µm), and ultracentrifuged at 100,000–120,000 × g for 70 min; pellets were washed once and resuspended in PBS.

Vesicle identity was confirmed by TEM (negative staining; cup-shaped 50–150 nm particles), NTA (modal size ∼100 nm), and immunoblotting for TSG101, CD63, HSP70 (with calnexin as a negative control when indicated). For uptake assays, exosomes were labeled with DiI/PKH, free dye was removed by re-isolation, and internalization by recipient cells was visualized by fluorescence microscopy. All spins were performed at 4 °C; dye-only and vehicle controls were included where relevant. Exosome isolation and characterization procedures were conducted in accordance with the Minimal Information for Studies of Extracellular Vesicles (MISEV2023) guidelines.

### 2.4 RNA extraction and quantitative real-time PCR

Total RNA (including miRNAs) was extracted from exosomes and cells using TRIzol/TRIzol-LS with DNase I treatment. Reverse transcription employed a stem-loop miRNA cDNA kit, and qRT-PCR was performed with SYBR Green chemistry on an ABI 7500 system. Each sample was run in technical triplicate with no-template and RT-blank controls. Relative expression was calculated by 2^^−ΔΔCt^. U6 snRNA served as the internal control for cellular assays. For exosomal miRNA assays, a synthetic spike-in control (cel-miR-39) was used for normalization.

### 2.5 Western blotting

Cells or exosome pellets were lysed in RIPA buffer supplemented with protease and phosphatase inhibitors, clarified by centrifugation, and quantified by Bicinchoninic Acid (BCA) assay. Equal protein amounts (20–30 µg for cells; 10–20 µg for exosomes) were separated by odium dodecyl sulfate–polyacrylamide gel electrophoresis (SDS–PAGE) and transferred to polyvinylidene difluoride (PVDF) membranes. After blocking in 5% non-fat milk, membranes were incubated overnight at 4 °C with primary antibodies against exosomal markers (TSG101, CD63, HSP70; and, where indicated, negative control calnexin), endothelial nitric oxide synthase (eNOS) and phospho-eNOS, Hippo pathway proteins (MST1, LATS1, phospho-LATS1, YAP, phospho-YAP), and apoptosis-related proteins (Cleaved Caspase-3, Bax, Bcl-2), followed by horseradish peroxidase (HRP)-conjugated secondary antibodies. Bands were visualized by enhanced chemiluminescence and quantified in ImageJ; phospho-proteins were normalized to their total protein and all targets to GAPDH. Inclusion of calnexin as a negative exosomal marker and tetraspanins as positives followed established practice in endothelial- and fibroblast-derived exosome studies.

### 2.6 Cell culture, treatments, and transfection

Human cardiac microvascular endothelial cells (HCMECs) were maintained in endothelial growth medium (10% exosome-depleted FBS, 1% penicillin/streptomycin) at 37 °C in 5% CO_2_. To model hyperglycemia, cells were exposed to high glucose (HG, 40 mM) for 48 h; normal glucose (NG, 5.5 mM) served as control. Where indicated, cells were transfected with miR-550a-5p inhibitor or negative control (Lipofectamine 3,000) for 24–48 h prior to assays. To interrogate Hippo signaling, the MST1/2 inhibitor XMU-MP-1 (which reduces YAP phosphorylation) was applied at the indicated concentrations and durations. AC16 human cardiomyocytes were cultured in DMEM/F12 with 10% FBS and used for exosome uptake and injury assays. Culture in exosome-depleted FBS and HG/NG conditions, as well as timelines for conditioned-medium collection in endothelial systems, followed prior exosome work in diabetic cardiomyopathy.

### 2.7 Preparation of exosomes for cell treatment and labeling/uptake assays

For mechanistic experiments, exosomes isolated from HCMEC supernatants under NG/HG ± transfection were quantified by BCA and added to recipient AC16 cells at a defined protein dose (10 μg/mL) for 24 h, consistent with dose ranges validated in endothelial–cardiac exosome studies. The same protein-normalized dose was applied across all exosome treatment groups to ensure comparability. For tracking, vesicles were stained with PKH26/PKH67 according to the manufacturer’s instructions, re-isolated to remove free dye, and incubated with AC16 cells (4 h). Cells were fixed with 4% paraformaldehyde (PFA), and nuclei were counterstained with 4′,6-diamidino-2-phenylindole (DAPI) and images acquired on a fluorescence/confocal microscope to confirm internalization. Dye-only “mock-labeled” controls were processed in parallel to exclude artifactual signals.

### 2.8 Functional assays in HCMECs

Cell viability was quantified by the Cell Counting Kit-8 (CCK-8) assay. Briefly, cells were seeded at standardized densities in 96-well plates, treated as indicated (NG/HG, miRNA inhibitor, XMU-MP-1), incubated with CCK-8 reagent for 1 h, and absorbance was read at 450 nm. Transwell migration was assessed using 8 µm pore inserts; after 24 h, migrated cells on the lower membrane were fixed, crystal-violet–stained, imaged, and counted. Tube formation was evaluated on growth-factor-reduced Matrigel; pre-treated HCMECs were seeded onto polymerized Matrigel and capillary-like structures quantified (total tube length, branch points) after 6 h using ImageJ/Angiogenesis Analyzer. These procedures, including readouts and quantification schemes, were aligned with recently reported endothelial exosome studies.

### 2.9 Functional assays in AC16 cardiomyocytes

After exosome exposure (N-Exo, H-Exo, H-NC-Exo, H-in-miR-Exo), cell viability (CCK-8) and apoptosis (TUNEL staining) were assessed according to kit instructions; TUNEL-positive nuclei were expressed as a percentage of DAPI-positive nuclei. Apoptosis signaling was further evaluated by immunoblotting for Cleaved Caspase-3, Bax, and Bcl-2. Cytotoxicity was quantified by measuring LDH release in culture supernatants. Oxidative stress endpoints included intracellular ROS (DCFH-DA fluorescence), MDA (thiobarbituric acid reactive substances), and SOD activity (xanthine oxidase method). Experimental schedules mirrored endothelial-to-cardiomyocyte exosome transfer paradigms described in diabetic cardiomyopathy literature.

### 2.10 Statistical analysis

Continuous variables were expressed as mean ± SD (or median [IQR] where appropriate). Normality of continuous variables was assessed using the Shapiro–Wilk test. Between-group comparisons used two-tailed Student’s t-test or Mann–Whitney U test; multi-group data were analyzed by one-way ANOVA with Tukey’s post hoc test or Kruskal–Wallis with Dunn’s correction. Spearman rank correlation assessed associations between miRNA levels and myocardial injury biomarkers. Multivariable logistic regression (forward stepwise; covariates selected *a priori* based on clinical relevance and univariable screening) evaluated whether candidate miRNAs independently predicted microvascular dysfunction; model diagnostics included collinearity checks and goodness-of-fit. ROC curves estimated diagnostic performance, with AUCs compared by DeLong’s test and 95% CIs obtained by bootstrap resampling (1,000 iterations). Multiple testing was controlled using Benjamini–Hochberg FDR where indicated. Analyses were performed in R (v4.x) and GraphPad Prism; *P* < 0.05 (two-sided) denoted statistical significance. Where relevant, reporting adhered to MIQE and EV-TRACK recommendations that are widely adopted in exosome research.

## 3 Results

### 3.1 Identification of differentially expressed miRNAs and key candidates

**FIGURE 1:**
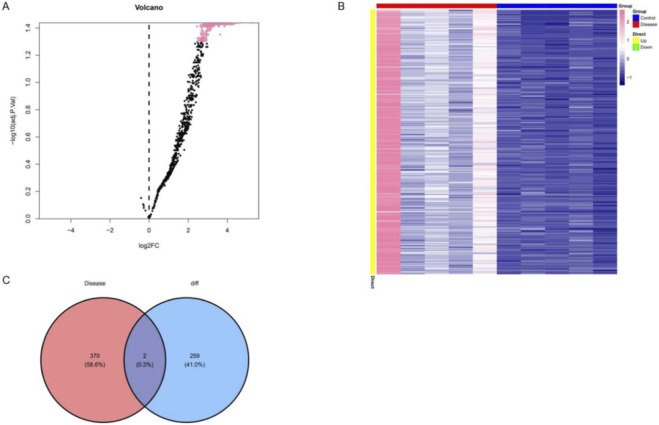
Identification of differentially expressed circulating exosomal miRNAs in diabetic patients with microvascular dysfunction. **(A)** Volcano plot showing differentially expressed circulating exosomal miRNAs between diabetic patients with and without microvascular dysfunction. **(B)** Heatmap of significantly differentially expressed miRNAs, with samples grouped according to microvascular dysfunction status. **(C)** Venn diagram showing the overlap between differentially expressed circulating exosomal miRNAs identified in this study and microvascular dysfunction–associated miRNAs curated from public databases.

Differential expression analysis between diabetic patients with and without microvascular dysfunction identified 264 significantly dysregulated miRNAs. The distribution of upregulated and downregulated miRNAs was visualized using a volcano plot (Figure 1A), while hierarchical clustering heatmap highlighted distinct expression patterns across samples, indicating clear separation between groups (Figure 1B).

To further refine candidate molecules, a total of 472 disease-associated miRNAs were retrieved from the HMDD and miRNet databases. The intersection with differentially expressed miRNAs resulted in the identification of two key miRNAs: hsa-miR-550a-5p and hsa-miR-665 (Figure 1C). Target gene prediction was subsequently performed using both miRDB and TargetScan databases, yielding 126 predicted targets for hsa-miR-550a-5p and 185 predicted targets for hsa-miR-665, with the complete gene lists provided in Supplementary Tables S1, S2, which formed the basis for downstream functional analyses.

### 3.2 Bioinformatic analyses of candidate miRNAs and regulatory networks

**FIGURE 2:**
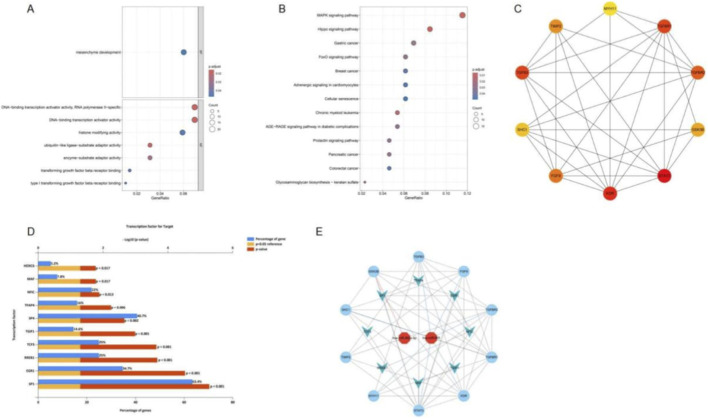
Functional enrichment analysis, hub gene network construction, transcription factor prediction, and integrated miRNA–gene–TF network. **(A)** GO enrichment bubble plot of target genes regulated by candidate miRNAs. **(B)** KEGG pathway enrichment bubble plot. **(C)** Protein–protein interaction (PPI) network of predicted target genes, with nodes representing genes and edges indicating protein interactions. **(D)** Transcription factors predicted to be associated with the predicted target genes, based on enrichment analysis. **(E)** Integrated miRNA–gene–transcription factor (TF) network constructed from candidate miRNAs, predicted target genes, and enriched TFs.

To explore the functional relevance of the predicted target genes, GO and KEGG enrichment analyses were conducted. GO analysis revealed significant enrichment in categories such as mesenchyme development, TGF-β receptor binding, histone modification activity, and transcription activator activity (RNA polymerase II–specific) (Figure 2A). KEGG pathway analysis demonstrated enrichment in multiple signaling pathways, including the MAPK, Hippo, and FoxO signaling pathways, as well as several cancer-related pathways (e.g., breast, colorectal, and pancreatic cancer). Notably, enrichment was also observed in cellular senescence and the AGE–RAGE signaling pathway, which are closely associated with diabetic complications (Figure 2B).

A protein–protein interaction (PPI) network was constructed based on STRING data, and the top 10 hub genes were identified using MCC ranking (Figure 2C). In addition, transcription factor (TF) prediction using FunRich identified 193 TFs associated with the predicted target genes, with the top 10 including HOXC6, MAF, NFIC, TFAP4, SP1, SP4, TGIF1, TCF3, RREB1, and EGR1 (Figure 2D). Finally, an integrated miRNA–gene–TF regulatory network was established, comprising two key miRNAs, 10 hub genes, and eight core transcription factors, thereby delineating the potential regulatory landscape underlying diabetes-associated microvascular dysfunction (Figure 2E).

### 3.3 Clinical characteristics and expression of candidate miRNAs

Baseline characteristics of participants are summarized in Table 1. Overall, there were no significant differences in demographic variables (age, sex, BMI, prevalence of hypertension and dyslipidemia) between the MD and NMD groups. However, patients with microvascular dysfunction exhibited a longer duration of diabetes and higher HbA1c levels, consistent with more severe metabolic impairment. In addition, the MD group showed elevated levels of myocardial injury markers (TnT, TnI, NT-proBNP, and CK-MB), particularly in the MD-MI subgroup, supporting the clinical relevance of the grouping strategy.

**TABLE 1:** Baseline clinical characteristics of patients with and without coronary microvascular dysfunction.

Variables	NMD group (n = 62)	MD group (n = 100)	χ^2^/t	P
Age (years)	54.53 ± 7.43	55.73 ± 8.76	−0.895	0.372
Sex (%)	​	​	0.002	0.962
Male	30 (48.39)	48 (48.00)	​	​
Female	32 (51.61)	52 (52.00)	​	​
BMI (kg/m^2^)	25.65 ± 4.31	25.57 ± 3.39	0.135	0.893
Duration of diabetes (years)	9.81 ± 3.92	11.18 ± 3.69	−2.21	0.037
Hypertension (%)	​	​	2.84	0.092
No	40 (64.52)	51 (51.00)	​	​
Yes	22 (35.48)	49 (49.00)	​	​
Dyslipidemia (%)	​	​	0.048	0.827
No	38 (61.29)	63 (63.00)	​	​
Yes	24 (38.71)	37 (37.00)	​	​
Antihypertensive medication use (%)	​	​	3.134	0.077
No	42 (67.74)	52 (52.00)	​	​
Yes	20 (32.26)	48 (48.00)	​	​
Statin use (%)	​	​	0.227	0.633
No	38 (61.29)	63 (63.00)	​	​
Yes	24 (38.71)	37 (37.00)	​	​
Oral glucose-lowering drug use (%)	​	​	0.088	0.767
No	30 (48.39)	46 (46.00)	​	​
Yes	32 (51.61)	54 (54.00)	​	​
HbA1c (%)	7.60 ± 1.41	8.16 ± 1.38	−2.801	0.014
TnT	11.57 ± 4.99	14.41 ± 5.21	−3.431	0.001
TnI	7.62 ± 3.28	8.92 ± 3.55	−2.332	0.021
NT-proBNP	137.42 ± 60.35	161.95 ± 74.55	−1.982	0.056
CK-MB	19.38 ± 6.81	22.33 ± 7.48	−2.527	0.012
hsa-miR-550a-5p	1.12 ± 0.55	2.05 ± 0.89	−7.436	<0.001
hsa-miR-665	1.09 ± 0.43	2.00 ± 1.00	−6.769	<0.001

**FIGURE 3:**
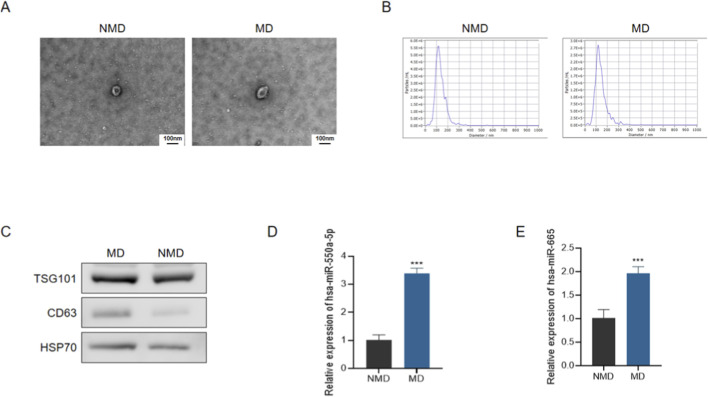
Characterization of circulating exosomes and expression of candidate miRNAs in diabetic patients with microvascular dysfunction. **(A)** Representative transmission electron microscopy (TEM) images of plasma-derived exosomes from patients without microvascular dysfunction (NMD) and with microvascular dysfunction (MD). Scale bar = 100 nm. **(B)** Representative nanoparticle tracking analysis (NTA) size distribution profiles of circulating exosomes. **(C)** Representative Western blot analysis of exosomal marker proteins (TSG101, CD63, and HSP70). **(D,E)** qRT-PCR analysis of circulating exosomal hsa-miR-550a-5p **(D)** and hsa-miR-665 **(E)** expression in plasma-derived exosomes. Data are presented as mean ± SD. ^***^ *P* < 0.001 vs. NMD.

To validate the presence and purity of circulating exosomes, transmission electron microscopy (TEM) demonstrated the typical cup-shaped morphology of extracellular vesicles in both groups (Figure 3A). Nanoparticle tracking analysis (NTA) further confirmed that the majority of vesicles had a diameter of ∼100 nm, consistent with the expected size distribution of exosomes (Figure 3B). The expression of canonical exosomal markers (TSG101, CD63, and HSP70) was verified by Western blotting, confirming successful isolation of exosomes (Figure 3C).

Subsequently, qRT-PCR analysis revealed that both *hsa-miR-550a-5p* and *hsa-miR-665* were significantly upregulated in circulating exosomes from the MD group compared with the NMD group (Figures 3D,E, *P* < 0.001). These findings suggest that exosomal miR-550a-5p and miR-665 may serve as potential biomarkers for microvascular dysfunction in diabetes.

### 3.4 Diagnostic and clinical relevance of candidate miRNAs

**FIGURE 4:**
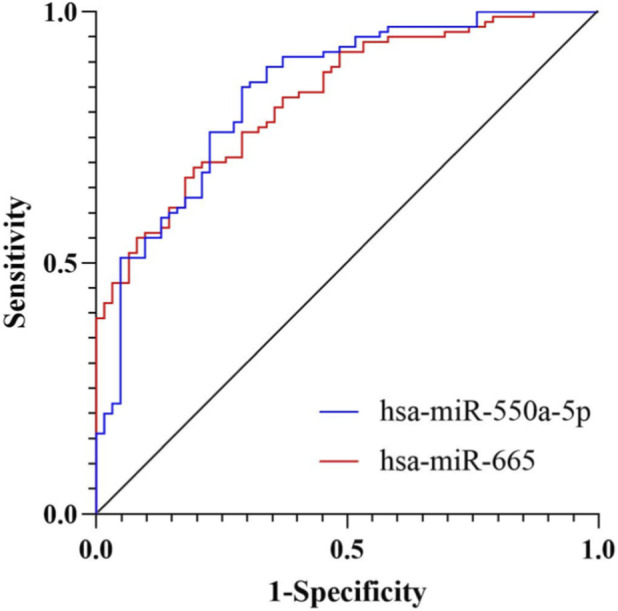
ROC curve analysis of candidate miRNAs for diabetic microvascular dysfunction. ROC curves showing the diagnostic performance of exosomal hsa-miR-550a-5p and hsa-miR-665 in discriminating MD from NMD. The AUC for hsa-miR-550a-5p was 0.840 (95% CI: 0.777–0.903), and that for hsa-miR-665 was 0.829 (95% CI: 0.768–0.890).

To assess the diagnostic potential of circulating exosomal miRNAs, receiver operating characteristic (ROC) curve analyses were performed. The area under the curve (AUC) for exosomal hsa-miR-550a-5p was 0.840 (95% CI: 0.777–0.903), and that for hsa-miR-665 was 0.829 (95% CI: 0.768–0.890) (both P < 0.001), indicating good discriminatory performance for diabetic microvascular dysfunction (Figure 4).

**FIGURE 5:**
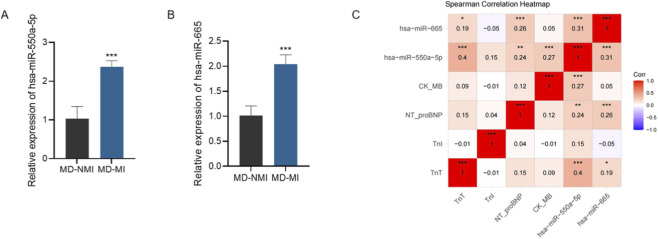
Expression and clinical relevance of candidate miRNAs in myocardial injury. Expression and clinical relevance of candidate miRNAs in myocardial injury. **(A,B)** qRT-PCR analysis of circulating exosomal hsa-miR-550a-5p **(A)** and hsa-miR-665 **(B)** expression in patients with microvascular dysfunction with myocardial injury (MD-MI) and without myocardial injury (MD-NMI). Data are presented as mean ± SD. ^***^ *P* < 0.001 vs. MD-NMI. **(C)** Spearman correlation heatmap showing associations between candidate miRNAs and cardiac injury markers, including TnT, TnI, NT-proBNP, and CK-MB.

We further investigated the association between candidate miRNAs and myocardial injury. Expression analysis revealed that both hsa-miR-550a-5p and hsa-miR-665 were significantly upregulated in patients with microvascular dysfunction and concomitant myocardial injury (MD-MI) compared with those without myocardial injury (MD-NMI) (Figures 5A,B, *P* < 0.001).

Moreover, Spearman correlation analyses demonstrated significant positive correlations between the expression of both miRNAs and serum cardiac injury biomarkers, including TnT, TnI, NT-proBNP, and CK-MB (Figure 5C). These results suggest that circulating exosomal miR-550a-5p and miR-665 not only serve as potential diagnostic biomarkers for microvascular dysfunction, but also reflect the severity of myocardial injury in diabetic patients.

### 3.5 Functional role of miR-550a-5p in endothelial cells

**FIGURE 6:**
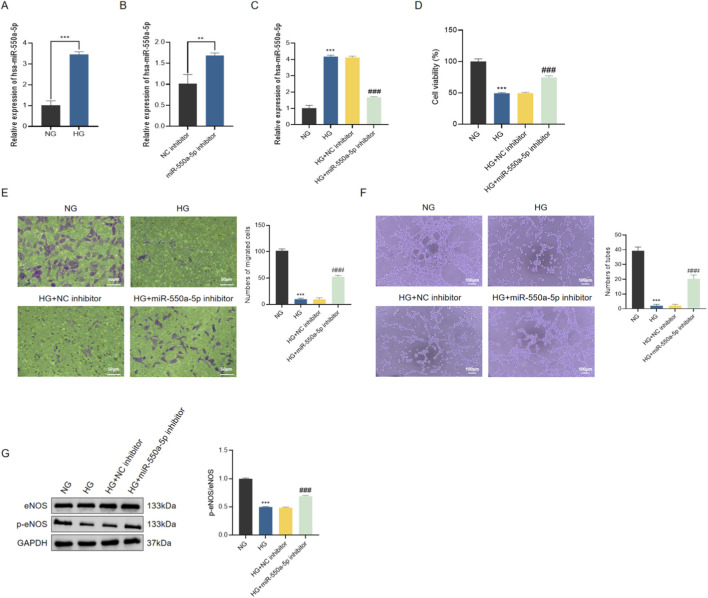
miR-550a-5p is upregulated in HG-treated HCMECs and promotes endothelial dysfunction. **(A)** qRT-PCR analysis of miR-550a-5p expression in NG vs. HG groups. **(B,C)** Verification of miR-550a-5p knockdown by inhibitor transfection in HG-treated HCMECs. **(D)** CCK-8 assay showing decreased cell viability in HG-treated cells, rescued by miR-550a-5p inhibition. **(E)** Transwell migration assay results scale bar = 50 µm. **(F)** Tube formation assay results scale bar = 100 µm. **(G)** Western blot analysis of eNOS and p-eNOS levels; bar graph shows p-eNOS/eNOS ratio. Data are presented as mean ± SD from three independent experiments (n = 3). ^***^ *P* < 0.001 vs. NG; ^###^ *P* < 0.001 vs. HG.

To further validate the role of miR-550a-5p in endothelial dysfunction, human cardiac microvascular endothelial cells (HCMECs) were cultured under normal glucose (NG) and high glucose (HG) conditions. qRT-PCR confirmed that miR-550a-5p expression was significantly upregulated in HG-treated HCMECs compared with NG controls (Figure 6A). Transfection with a miR-550a-5p inhibitor effectively suppressed its expression (Figures 6B,C).

Functionally, HG exposure markedly reduced HCMEC viability, migratory capacity, and tube formation ability, as assessed by CCK-8 assay, Transwell migration, and Matrigel tube formation assays, respectively. Inhibition of miR-550a-5p significantly alleviated these impairments (Figures 6D–F). Consistently, Western blot analysis showed that HG treatment led to a decrease in the ratio of phosphorylated eNOS to total eNOS (p-eNOS/eNOS), which was partially restored by miR-550a-5p inhibition (Figure 6G). These findings indicate that miR-550a-5p contributes to HG-induced endothelial dysfunction by impairing NO bioavailability and angiogenic capacity.

**FIGURE 7:**
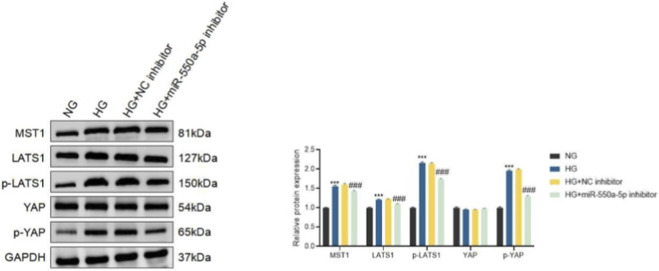
miR-550a-5p modulates Hippo signaling in HCMECs under high-glucose conditions. Western blot analysis of Hippo pathway components (MST1, LATS1, p-LATS1, YAP, and p-YAP) in normal glucose (NG), high glucose (HG), HG plus negative control inhibitor (HG + NC inhibitor), and HG plus miR-550a-5p inhibitor (HG + miR-550a-5p inhibitor)–treated HCMECs. Right panels show densitometric quantification normalized to GAPDH or total protein as appropriate. Data are presented as mean ± SD from three independent experiments (n = 3). ^***^ *P* < 0.001 vs. NG; ^###^ *P* < 0.001 vs. HG.

Given the enrichment analysis implicating the Hippo signaling pathway, we next examined its activation status. HG stimulation resulted in the upregulation of MST1 and LATS1, accompanied by increased phosphorylation of LATS1 and YAP, indicative of Hippo pathway activation (Figure 7). Importantly, miR-550a-5p inhibition significantly attenuated these changes, suggesting that miR-550a-5p is functionally associated with activation of Hippo signaling and endothelial dysfunction under high-glucose conditions.

To further confirm the involvement of Hippo–YAP signaling, we employed the MST1/2 inhibitor XMU-MP-1 (a Hippo pathway inhibitor that reduces YAP phosphorylation). Treatment with XMU-MP-1 alone partially restored endothelial cell viability, migration, tube formation, and p-eNOS/eNOS levels in HG-treated HCMECs, while combined treatment with miR-550a-5p inhibition and XMU-MP-1 produced the most pronounced rescue effects (Supplementary Figures S1B–E). At the molecular level, both miR-550a-5p inhibition and XMU-MP-1 suppressed HG-induced upregulation of MST1, LATS1, p-LATS1, and p-YAP, while enhancing YAP expression; the combination resulted in maximal suppression of Hippo pathway activation (Supplementary Figure S1A). These findings support the involvement of Hippo–YAP signaling in miR-550a-5p–associated endothelial dysfunction under high glucose, based on pharmacologic pathway modulation and concordant changes in pathway readouts.

To further elucidate the involvement of the Hippo pathway, we examined whether inhibition of YAP phosphorylation could enhance the protective effects of miR-550a-5p silencing. Treatment with the selective YAP inhibitor XMU-MP-1 significantly reduced Hippo pathway activation, as reflected by decreased levels of MST1, LATS1, p-LATS1, and p-YAP, while concomitantly restoring YAP expression (Supplementary Figure S1A). Functionally, both miR-550a-5p inhibition and XMU-MP-1 treatment partially restored cell viability, migration, and tube formation capacity in HG-treated HCMECs (Supplementary Figures S1B–D). Notably, combined treatment produced the most pronounced rescue effects, suggesting a synergistic interaction. Western blot analysis further confirmed that the combination of miR-550a-5p inhibition and XMU-MP-1 restored the p-eNOS/eNOS ratio more effectively than either treatment alone (Supplementary Figure S1E). These findings strongly support the notion that miR-550a-5p promotes endothelial dysfunction through Hippo pathway–dependent mechanisms.

### 3.6 Exosome-mediated transfer of miR-550a-5p from HCMECs to cardiomyocytes

To determine whether miR-550a-5p can be encapsulated in endothelial exosomes and transferred into cardiomyocytes, we first performed a comprehensive characterization of HCMEC-derived exosomes. Transmission electron microscopy (TEM) revealed round or cup-shaped vesicles with a lipid bilayer membrane in all groups (N-Exo, H-Exo, H-NC-Exo, H-in-miR-Exo), consistent with the typical morphology of exosomes (Supplementary Figure S2A). Nanoparticle tracking analysis (NTA) showed that vesicle sizes ranged between 55 and 175 nm, with peak diameters around 100 nm, further confirming their exosomal nature (Supplementary Figure S2B). In addition, Western blotting confirmed the enrichment of exosomal markers TSG101, CD63, and HSP70 across all groups (Supplementary Figure S2C). These results validated the successful isolation of high-purity exosomes from HCMECs.

**FIGURE 8:**
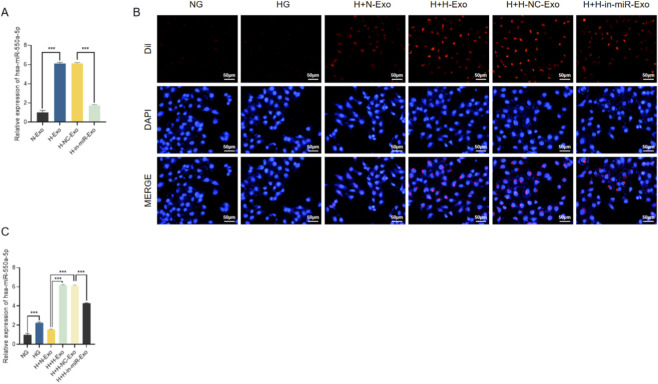
HCMEC-derived exosomes mediate the transfer of miR-550a-5p to AC16 cardiomyocytes. **(A)** qRT-PCR analysis of miR-550a-5p levels in exosomes from different HCMEC treatment groups. **(B)** Immunofluorescence showing Dil-labeled exosomes internalized by AC16 cells. Scale bar = 50 µm. **(C)** qRT-PCR analysis of miR-550a-5p expression in AC16 cells after exosome co-culture. Data are presented as mean ± SD from three independent experiments (n = 3). ^***^ *P* < 0.001 compared with the indicated control group.

We next evaluated the levels of miR-550a-5p in exosomes derived from different conditions. qRT-PCR analysis revealed that H-Exo and H-NC-Exo contained significantly higher levels of miR-550a-5p compared with N-Exo, whereas H-in-miR-Exo (derived from HG-treated HCMECs transfected with miR-550a-5p inhibitor) showed markedly reduced miR-550a-5p expression (Figure 8A). To assess exosome uptake, Dil-labeled exosomes were co-cultured with AC16 cardiomyocytes. Red fluorescence signals were readily detected in AC16 cells treated with N-Exo, H-Exo, or H-NC-Exo, whereas the NG control showed minimal signal, confirming efficient internalization of exosomes by recipient cardiomyocytes (Figure 8B). Concordantly, qRT-PCR demonstrated that intracellular levels of miR-550a-5p were significantly elevated in AC16 cells after incubation with H-Exo and H-NC-Exo, but only slightly increased with N-Exo, while H-in-miR-Exo failed to induce miR-550a-5p upregulation (Figure 8C). Together, these findings indicate that HCMEC-derived exosomes act as carriers, transferring miR-550a-5p into cardiomyocytes.

**FIGURE 9:**
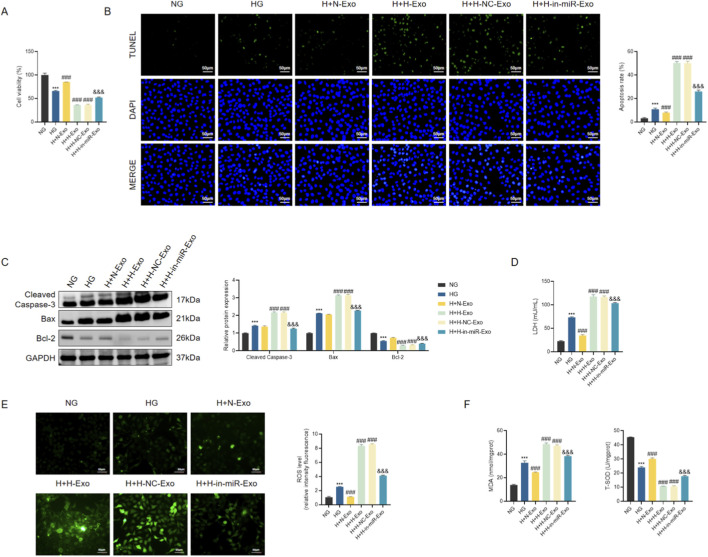
Exosomal miR-550a-5p exacerbates myocardial injury under hyperglycemic conditions. **(A)** CCK-8 assay of AC16 cell viability. **(B)** TUNEL staining showing apoptotic nuclei in AC16 cells. Scale bar = 50 µm. **(C)** Western blot analysis of apoptosis-related proteins (Cleaved Caspase-3, Bax, Bcl-2). **(D)** LDH release levels in culture supernatants. **(E)** ROS levels in AC16 cells. Scale bar = 50 µm. **(F)** Oxidative stress markers: MDA concentration and SOD activity. Data are presented as mean ± SD from three independent experiments (n = 3). ^**^ *P* < 0.001 vs. NG; ^##^ *P* < 0.001 vs. HG; ^&&^ *P* < 0.001 vs. H + H-NC-Exo.

We further explored the functional impact of exosomal miR-550a-5p on AC16 cardiomyocytes. Cell viability assays (CCK-8) revealed that HG-treated AC16 cells exhibited a significant decline in viability compared with NG controls. Co-culture with H-Exo or H-NC-Exo further aggravated this impairment, whereas N-Exo partially alleviated the effect. Strikingly, H-in-miR-Exo significantly restored cell viability, indicating that depletion of miR-550a-5p in exosomes mitigates HG-induced injury (Figure 9A).

Consistent with these results, apoptosis assays demonstrated pronounced cell death in H-Exo and H-NC-Exo groups. TUNEL staining revealed markedly increased numbers of apoptotic nuclei, accompanied by elevated protein expression of Cleaved Caspase-3 and Bax and reduced levels of the anti-apoptotic protein Bcl-2 (Figures 9B,C). In contrast, H-in-miR-Exo significantly decreased apoptosis rates, with molecular profiles shifting toward a protective phenotype.

Indicators of cellular injury and stress were also assessed. LDH release was significantly elevated in HG-treated AC16 cells, further increased in H-Exo and H-NC-Exo groups, but substantially reduced in the H-in-miR-Exo group (Figure 9D). Similarly, oxidative stress markers showed parallel trends: ROS and MDA levels were highest in H-Exo and H-NC-Exo, while SOD activity was lowest, indicating enhanced oxidative damage (Figures 9E,F). Notably, H-in-miR-Exo reversed these effects, reducing ROS and MDA while restoring SOD activity toward normal levels.

Collectively, these findings demonstrate that exosomes derived from HG-treated HCMECs deliver miR-550a-5p into cardiomyocytes, thereby promoting apoptosis, oxidative stress, and loss of cell viability. Silencing miR-550a-5p in endothelial exosomes abrogates these detrimental effects, highlighting a critical mechanism of endothelial–cardiomyocyte crosstalk in diabetic conditions.

## 4 Discussion

Taken together, our findings identify circulating exosomal miR-550a-5p together with miR-665 as clinically actionable biomarkers of endothelial–myocyte coupling in diabetes. At the systems level, these signals align with the HFpEF paradigm, in which coronary MD represents a mechanistically upstream process contributing to myocardial remodeling and symptom development. This may help explain why conventional metabolic indices underperform for early risk detection (Yeo et al., 2022). At the molecular level, our results integrate two established EV-miRNA threads introduced in the Introduction: (i) vascular EV-miRNAs such as the miR-30 family rise early with oxidative injury, endothelial senescence, and coronary rarefaction—before diastolic impairment—underscoring their value as subclinical sentinels of MD (Veitch et al., 2022); and (ii) restoring endothelial miRNA programs (e.g., exercise-induced miR-126) is necessary to preserve coronary structure and cardiac performance in experimental diabetes (Lew et al., 2020). Critically, our endothelial-to-cardiomyocyte transfer experiments indicate that endothelial-derived exosomal miR-550a-5p is functionally associated with Hippo–YAP pathway activity, analogous to previously reported exosomal Hippo-related cargo such as Mst1, supporting a mechanistically plausible link between microvascular stress and cardiomyocyte vulnerability, while acknowledging that direct miRNA–mRNA targeting and *in vivo* validation remain to be established (Hu et al., 2018). Mechanistically, our data indicate that miR-550a-5p is associated with coordinated changes in Hippo–YAP signaling and eNOS phosphorylation in microvascular endothelium under high-glucose conditions. This observation is consistent with broader evidence that Hippo pathway nodes interface with non-coding RNA networks regulating cardiac growth and stress responses (e.g., lncExACT1–DCHS2), and that microRNAs can modulate multiple Hippo components in injury-related contexts (Li et al., 2022; Lin et al., 2013).

Clinically, pairing exosomal miR-550a-5p/miR-665 with microvascular function phenotyping could enhance early stratification in T2DM, complementing hemodynamic and perfusion markers linked to MD and remodeling—such as ambulatory blood pressure associations in asymptomatic T2DM and the inverse relation between microvascular disease burden and cardiorespiratory fitness (Kaze et al., 2024; Yeo et al., 2022). Beyond diagnostics, feasibility signals from EV-based therapeutics—including donor-cell conditioning that reprograms vesicular miRNA cargo to boost endothelial repair—support the notion that antagomiR-550a-5p strategies or nodal Hippo modulation could be developed as microvascular-centric cardioprotection (Fan et al., 2025). Implementation will hinge on rigorous EV workflows (isolation, normalization, and reporting per MISEV; attention to EV heterogeneity and lipoprotein carryover), external validation across centers, longitudinal kinetics to establish temporal priority over MD, and unbiased target-network mapping to resolve how miR-550a-5p intersects with fatty-acid metabolism/ROS/eNOS circuitry emphasized by prior EV-miRNA studies (Veitch et al., 2022; Liu et al., 2024; Welsh et al., 2024; Thery et al., 2018). Notably, new coronary evidence linking exosomal miRNAs to endothelial fatty-acid degradation (e.g., miR-16-2-3p→ACADM) reinforces lipid-handling pathways as a shared, actionable substrate connecting endothelial fitness to myocardial resilience in diabetes. In aggregate, these findings indicate that circulating exosomal miRNAs are not merely passive correlates but reflect biologically relevant information associated with the endothelial–myocyte axis, supporting their potential use for earlier detection, dynamic monitoring, and mechanism-guided intervention in diabetic heart disease.

Several limitations warrant consideration. The single-centre, cross-sectional and retrospective design limits generalizability and precludes establishing temporal priority of exosomal miR-550a-5p/miR-665 relative to MD and myocardial injury, supporting the need for pre-registered, multi-centre prospective studies with external validation, consistent with TRIPOD + AI guidance (Collins et al., 2024). The bioinformatic discovery relied on a small public GEO dataset without batch annotations, and thus exploratory analyses may be affected by small-sample bias or residual batch effects. In addition, incomplete adjustment for medication classes or dosages may result in residual confounding. MD diagnosis was based on institutional clinical criteria rather than quantitative microvascular imaging, introducing potential phenotype heterogeneity or misclassification. In EV analyses, vesicle heterogeneity, co-isolated lipoproteins, and pre-analytical variability may bias miRNA attribution, underscoring the need for MISEV2023-compliant isolation, quantitative particle/protein metrics, appropriate positive and negative markers (e.g., ApoA1/ApoB) (Welsh et al., 2024) and exRNAQC-recommended controls (ex, 2025). Mechanistic inference is further constrained by *in vitro* models, as AC16 cells incompletely reflect adult cardiomyocyte physiology; validation in hiPSC-derived systems and genetic interrogation of Hippo–YAP signaling are warranted (Mitchell et al., 2020). Future studies integrating serial sampling with direct coronary microvascular phenotyping (e.g., CMR/PET flow reserve) (Kaze et al., 2024; Yeo et al., 2022), together with *in vivo* modulation and interventional designs, will be required to establish temporal priority, causality, and translational relevance.

## 5 Conclusion

Our data identify exosomal miR-550a-5p together with miR-665 as a dual-utility signal in type 2 diabetes, acting as an accessible biomarker for coronary microvascular dysfunction and a candidate mediator of endothelial-to-cardiomyocyte communication, supported by *in vitro* findings implicating Hippo–YAP/eNOS signaling. This proposed mechanism is consistent with endothelial-to-myocyte exosomal signaling observed in diabetic cardiomyopathy. Clinically, pairing these miRNAs with direct microvascular phenotyping offers a path to refine risk stratification and dynamic monitoring, while mechanistically, targeting miR-550a-5p/Hippo nodes may help restore endothelial function and mitigate vesicle-borne injury signals—converging with evidence that normalization of endothelial miRNAs (for example, exercise-linked miR-126) preserves coronary and cardiac function in diabetes.

## Data Availability

The original contributions presented in the study are included in the article/Supplementary Material, further inquiries can be directed to the corresponding authors.
