# Supplementary material for: Circulating exosomal miR-550a-5p/miR-665 identify coronary microvascular dysfunction and drive endothelial–myocyte crosstalk in type 2 diabetes
Source: Front Cell Dev Biol. 2026 Jan 29;14:1744708. doi: 10.3389/fcell.2026.1744708 (PMC12894409; doi:10.3389/fcell.2026.1744708)
Supplement: Supplementary file 1 [file DataSheet1.docx]

| Target Rank | Target Score | miRNA Name | Gene Symbol | Gene Description |
| --- | --- | --- | --- | --- |
| 6 | 97 | hsa-miR-665 | KDR | kinase insert domain receptor |
| 9 | 95 | hsa-miR-665 | TP53I11 | tumor protein p53 inducible protein 11 |
| 11 | 94 | hsa-miR-665 | TRIM66 | tripartite motif containing 66 |
| 19 | 94 | hsa-miR-665 | KCNN3 | potassium calcium-activated channel subfamily N member 3 |
| 20 | 94 | hsa-miR-665 | HOXB5 | homeobox B5 |
| 21 | 94 | hsa-miR-665 | ATP8A2 | ATPase phospholipid transporting 8A2 |
| 22 | 93 | hsa-miR-665 | TXNL1 | thioredoxin like 1 |
| 23 | 93 | hsa-miR-665 | TRIM8 | tripartite motif containing 8 |
| 25 | 93 | hsa-miR-665 | TGFBR1 | transforming growth factor beta receptor 1 |
| 26 | 93 | hsa-miR-665 | IGFN1 | immunoglobulin-like and fibronectin type III domain containing 1 |
| 27 | 92 | hsa-miR-665 | ONECUT2 | one cut homeobox 2 |
| 30 | 92 | hsa-miR-665 | UNC5B | unc-5 netrin receptor B |
| 32 | 92 | hsa-miR-665 | NIPBL | NIPBL, cohesin loading factor |
| 34 | 91 | hsa-miR-665 | LUZP1 | leucine zipper protein 1 |
| 35 | 91 | hsa-miR-665 | POU2F1 | POU class 2 homeobox 1 |
| 41 | 90 | hsa-miR-665 | SLC37A3 | solute carrier family 37 member 3 |
| 42 | 90 | hsa-miR-665 | ZNF609 | zinc finger protein 609 |
| 43 | 90 | hsa-miR-665 | DOK6 | docking protein 6 |
| 44 | 90 | hsa-miR-665 | PRR14L | proline rich 14 like |
| 45 | 90 | hsa-miR-665 | COPS7B | COP9 signalosome subunit 7B |
| 46 | 90 | hsa-miR-665 | PSMF1 | proteasome inhibitor subunit 1 |
| 48 | 90 | hsa-miR-665 | SUMO1 | small ubiquitin-like modifier 1 |
| 49 | 89 | hsa-miR-665 | UBE2Q1 | ubiquitin conjugating enzyme E2 Q1 |
| 50 | 89 | hsa-miR-665 | HEYL | hes related family bHLH transcription factor with YRPW motif-like |
| 51 | 89 | hsa-miR-665 | KIF21B | kinesin family member 21B |
| 52 | 89 | hsa-miR-665 | RASGEF1B | RasGEF domain family member 1B |
| 56 | 88 | hsa-miR-665 | LASP1 | LIM and SH3 protein 1 |
| 58 | 88 | hsa-miR-665 | STX3 | syntaxin 3 |
| 60 | 87 | hsa-miR-665 | NLK | nemo like kinase |
| 61 | 87 | hsa-miR-665 | TGFBR2 | transforming growth factor beta receptor 2 |
| 69 | 86 | hsa-miR-665 | USP49 | ubiquitin specific peptidase 49 |
| 70 | 86 | hsa-miR-665 | ANKRD13C | ankyrin repeat domain 13C |
| 72 | 86 | hsa-miR-665 | CHKB | choline kinase beta |
| 75 | 86 | hsa-miR-665 | ALG8 | ALG8, alpha-1,3-glucosyltransferase |
| 87 | 85 | hsa-miR-665 | HABP4 | hyaluronan binding protein 4 |
| 90 | 85 | hsa-miR-665 | SLC46A1 | solute carrier family 46 member 1 |
| 94 | 85 | hsa-miR-665 | RIMS4 | regulating synaptic membrane exocytosis 4 |
| 98 | 84 | hsa-miR-665 | GDAP1L1 | ganglioside induced differentiation associated protein 1 like 1 |
| 102 | 84 | hsa-miR-665 | CARHSP1 | calcium regulated heat stable protein 1 |
| 105 | 83 | hsa-miR-665 | MLLT6 | MLLT6, PHD finger containing |
| 107 | 83 | hsa-miR-665 | SMAD7 | SMAD family member 7 |
| 114 | 82 | hsa-miR-665 | TIMP3 | TIMP metallopeptidase inhibitor 3 |
| 115 | 82 | hsa-miR-665 | MLLT1 | MLLT1, super elongation complex subunit |
| 118 | 82 | hsa-miR-665 | PKNOX2 | PBX/knotted 1 homeobox 2 |
| 119 | 82 | hsa-miR-665 | CYTH3 | cytohesin 3 |
| 121 | 81 | hsa-miR-665 | DLX3 | distal-less homeobox 3 |
| 125 | 81 | hsa-miR-665 | CHST3 | carbohydrate sulfotransferase 3 |
| 129 | 80 | hsa-miR-665 | SV2C | synaptic vesicle glycoprotein 2C |
| 130 | 80 | hsa-miR-665 | PRELP | proline and arginine rich end leucine rich repeat protein |
| 133 | 80 | hsa-miR-665 | FBXO41 | F-box protein 41 |
| 134 | 80 | hsa-miR-665 | ABHD4 | abhydrolase domain containing 4 |
| 145 | 79 | hsa-miR-665 | TBC1D16 | TBC1 domain family member 16 |
| 154 | 79 | hsa-miR-665 | PRSS8 | serine protease 8 |
| 157 | 79 | hsa-miR-665 | CDC25A | cell division cycle 25A |
| 158 | 79 | hsa-miR-665 | EXT1 | exostosin glycosyltransferase 1 |
| 168 | 78 | hsa-miR-665 | PPP2R2A | protein phosphatase 2 regulatory subunit Balpha |
| 173 | 77 | hsa-miR-665 | B3GNT7 | UDP-GlcNAc:betaGal beta-1,3-N-acetylglucosaminyltransferase 7 |
| 178 | 77 | hsa-miR-665 | SLC23A2 | solute carrier family 23 member 2 |
| 179 | 77 | hsa-miR-665 | ATG13 | autophagy related 13 |
| 183 | 77 | hsa-miR-665 | SRF | serum response factor |
| 186 | 77 | hsa-miR-665 | MMP24 | matrix metallopeptidase 24 |
| 188 | 76 | hsa-miR-665 | NME7 | NME/NM23 family member 7 |
| 193 | 76 | hsa-miR-665 | KCNB1 | potassium voltage-gated channel subfamily B member 1 |
| 197 | 76 | hsa-miR-665 | VPS4A | vacuolar protein sorting 4 homolog A |
| 200 | 76 | hsa-miR-665 | AKT3 | AKT serine/threonine kinase 3 |
| 202 | 76 | hsa-miR-665 | GRIK3 | glutamate ionotropic receptor kainate type subunit 3 |
| 208 | 75 | hsa-miR-665 | PRR13 | proline rich 13 |
| 210 | 75 | hsa-miR-665 | BTN3A2 | butyrophilin subfamily 3 member A2 |
| 214 | 75 | hsa-miR-665 | SEMA3G | semaphorin 3G |
| 239 | 73 | hsa-miR-665 | PACS1 | phosphofurin acidic cluster sorting protein 1 |
| 242 | 73 | hsa-miR-665 | CORO2B | coronin 2B |
| 246 | 73 | hsa-miR-665 | TMEM242 | transmembrane protein 242 |
| 247 | 73 | hsa-miR-665 | GLIPR2 | GLI pathogenesis related 2 |
| 249 | 73 | hsa-miR-665 | VPS53 | VPS53, GARP complex subunit |
| 250 | 73 | hsa-miR-665 | BCAM | basal cell adhesion molecule (Lutheran blood group) |
| 251 | 73 | hsa-miR-665 | MCRS1 | microspherule protein 1 |
| 255 | 73 | hsa-miR-665 | RPS6KA3 | ribosomal protein S6 kinase A3 |
| 260 | 72 | hsa-miR-665 | MYH11 | myosin heavy chain 11 |
| 264 | 72 | hsa-miR-665 | SMYD1 | SET and MYND domain containing 1 |
| 270 | 72 | hsa-miR-665 | SNX21 | sorting nexin family member 21 |
| 276 | 72 | hsa-miR-665 | ENO2 | enolase 2 |
| 281 | 71 | hsa-miR-665 | STAT3 | signal transducer and activator of transcription 3 |
| 290 | 71 | hsa-miR-665 | OVOL1 | ovo like transcriptional repressor 1 |
| 300 | 71 | hsa-miR-665 | NNAT | neuronatin |
| 302 | 70 | hsa-miR-665 | POLR3H | RNA polymerase III subunit H |
| 307 | 70 | hsa-miR-665 | PRICKLE2 | prickle planar cell polarity protein 2 |
| 320 | 70 | hsa-miR-665 | PGPEP1 | pyroglutamyl-peptidase I |
| 322 | 69 | hsa-miR-665 | ZNF710 | zinc finger protein 710 |
| 323 | 69 | hsa-miR-665 | SOX17 | SRY-box 17 |
| 330 | 69 | hsa-miR-665 | CACNB3 | calcium voltage-gated channel auxiliary subunit beta 3 |
| 336 | 69 | hsa-miR-665 | DPF2 | double PHD fingers 2 |
| 339 | 69 | hsa-miR-665 | ELN | elastin |
| 345 | 69 | hsa-miR-665 | OCEL1 | occludin/ELL domain containing 1 |
| 354 | 68 | hsa-miR-665 | RASSF7 | Ras association domain family member 7 |
| 355 | 68 | hsa-miR-665 | BBC3 | BCL2 binding component 3 |
| 358 | 68 | hsa-miR-665 | EHF | ETS homologous factor |
| 359 | 68 | hsa-miR-665 | SDC1 | syndecan 1 |
| 364 | 68 | hsa-miR-665 | RTN2 | reticulon 2 |
| 384 | 67 | hsa-miR-665 | ZNF618 | zinc finger protein 618 |
| 387 | 67 | hsa-miR-665 | NKIRAS2 | NFKB inhibitor interacting Ras like 2 |
| 393 | 67 | hsa-miR-665 | BTN2A2 | butyrophilin subfamily 2 member A2 |
| 397 | 67 | hsa-miR-665 | YBX2 | Y-box binding protein 2 |
| 400 | 66 | hsa-miR-665 | INSIG1 | insulin induced gene 1 |
| 404 | 66 | hsa-miR-665 | ADAM11 | ADAM metallopeptidase domain 11 |
| 407 | 66 | hsa-miR-665 | C11orf45 | chromosome 11 open reading frame 45 |
| 411 | 66 | hsa-miR-665 | SYT2 | synaptotagmin 2 |
| 414 | 66 | hsa-miR-665 | PAG1 | phosphoprotein membrane anchor with glycosphingolipid microdomains 1 |
| 417 | 66 | hsa-miR-665 | OCSTAMP | osteoclast stimulatory transmembrane protein |
| 418 | 66 | hsa-miR-665 | CACNG8 | calcium voltage-gated channel auxiliary subunit gamma 8 |
| 421 | 66 | hsa-miR-665 | CDC42SE1 | CDC42 small effector 1 |
| 426 | 66 | hsa-miR-665 | MEF2A | myocyte enhancer factor 2A |
| 427 | 66 | hsa-miR-665 | C3orf70 | chromosome 3 open reading frame 70 |
| 431 | 65 | hsa-miR-665 | PDGFRB | platelet derived growth factor receptor beta |
| 432 | 65 | hsa-miR-665 | NFATC4 | nuclear factor of activated T cells 4 |
| 439 | 65 | hsa-miR-665 | PALM | paralemmin |
| 444 | 65 | hsa-miR-665 | C6orf62 | chromosome 6 open reading frame 62 |
| 447 | 64 | hsa-miR-665 | PRR3 | proline rich 3 |
| 450 | 64 | hsa-miR-665 | GABRE | gamma-aminobutyric acid type A receptor epsilon subunit |
| 457 | 64 | hsa-miR-665 | LIX1L | limb and CNS expressed 1 like |
| 461 | 64 | hsa-miR-665 | PRR15L | proline rich 15 like |
| 465 | 64 | hsa-miR-665 | CABP7 | calcium binding protein 7 |
| 475 | 63 | hsa-miR-665 | PDE7A | phosphodiesterase 7A |
| 481 | 63 | hsa-miR-665 | FAM102A | family with sequence similarity 102 member A |
| 482 | 63 | hsa-miR-665 | CRIM1 | cysteine rich transmembrane BMP regulator 1 |
| 483 | 63 | hsa-miR-665 | P2RY2 | purinergic receptor P2Y2 |
| 488 | 62 | hsa-miR-665 | TAF5L | TATA-box binding protein associated factor 5 like |
| 497 | 62 | hsa-miR-665 | RIMKLB | ribosomal modification protein rimK like family member B |
| 504 | 62 | hsa-miR-665 | TMEM129 | transmembrane protein 129 |
| 511 | 61 | hsa-miR-665 | PAN2 | poly(A) specific ribonuclease subunit PAN2 |
| 515 | 61 | hsa-miR-665 | CAPN5 | calpain 5 |
| 518 | 61 | hsa-miR-665 | NKX3-2 | NK3 homeobox 2 |
| 533 | 61 | hsa-miR-665 | ST3GAL3 | ST3 beta-galactoside alpha-2,3-sialyltransferase 3 |
| 545 | 60 | hsa-miR-665 | ESRRG | estrogen related receptor gamma |
| 548 | 60 | hsa-miR-665 | CHURC1 | churchill domain containing 1 |
| 556 | 60 | hsa-miR-665 | MYO10 | myosin X |
| 558 | 60 | hsa-miR-665 | NAV1 | neuron navigator 1 |
| 571 | 59 | hsa-miR-665 | TMED9 | transmembrane p24 trafficking protein 9 |
| 574 | 59 | hsa-miR-665 | SPRED1 | sprouty related EVH1 domain containing 1 |
| 589 | 59 | hsa-miR-665 | ZNF512B | zinc finger protein 512B |
| 593 | 58 | hsa-miR-665 | FAM19A1 | family with sequence similarity 19 member A1, C-C motif chemokine like |
| 594 | 58 | hsa-miR-665 | B4GALNT1 | beta-1,4-N-acetyl-galactosaminyltransferase 1 |
| 601 | 58 | hsa-miR-665 | SEMA4A | semaphorin 4A |
| 603 | 58 | hsa-miR-665 | MTSS1L | MTSS1L, I-BAR domain containing |
| 615 | 58 | hsa-miR-665 | DIAPH1 | diaphanous related formin 1 |
| 619 | 58 | hsa-miR-665 | UQCC2 | ubiquinol-cytochrome c reductase complex assembly factor 2 |
| 628 | 57 | hsa-miR-665 | TMEM86A | transmembrane protein 86A |
| 632 | 57 | hsa-miR-665 | FGF9 | fibroblast growth factor 9 |
| 636 | 57 | hsa-miR-665 | TRMT61A | tRNA methyltransferase 61A |
| 648 | 57 | hsa-miR-665 | KLHDC3 | kelch domain containing 3 |
| 651 | 57 | hsa-miR-665 | KDM5C | lysine demethylase 5C |
| 656 | 57 | hsa-miR-665 | LDLRAD2 | low density lipoprotein receptor class A domain containing 2 |
| 658 | 57 | hsa-miR-665 | SRGAP2 | SLIT-ROBO Rho GTPase activating protein 2 |
| 665 | 56 | hsa-miR-665 | SURF4 | surfeit 4 |
| 668 | 56 | hsa-miR-665 | AJAP1 | adherens junctions associated protein 1 |
| 692 | 55 | hsa-miR-665 | C22orf46 | chromosome 22 open reading frame 46 |
| 695 | 55 | hsa-miR-665 | RAB3B | RAB3B, member RAS oncogene family |
| 696 | 55 | hsa-miR-665 | CRTC1 | CREB regulated transcription coactivator 1 |
| 702 | 55 | hsa-miR-665 | GRIN2B | glutamate ionotropic receptor NMDA type subunit 2B |
| 711 | 55 | hsa-miR-665 | PARVA | parvin alpha |
| 717 | 55 | hsa-miR-665 | MOCS1 | molybdenum cofactor synthesis 1 |
| 724 | 55 | hsa-miR-665 | TGFB3 | transforming growth factor beta 3 |
| 744 | 54 | hsa-miR-665 | SH2D4B | SH2 domain containing 4B |
| 750 | 54 | hsa-miR-665 | C1RL | complement C1r subcomponent like |
| 759 | 54 | hsa-miR-665 | UQCC1 | ubiquinol-cytochrome c reductase complex assembly factor 1 |
| 764 | 54 | hsa-miR-665 | AREL1 | apoptosis resistant E3 ubiquitin protein ligase 1 |
| 791 | 53 | hsa-miR-665 | GNL3L | G protein nucleolar 3 like |
| 795 | 53 | hsa-miR-665 | FAM160B2 | family with sequence similarity 160 member B2 |
| 802 | 53 | hsa-miR-665 | ADCYAP1R1 | ADCYAP receptor type I |
| 803 | 53 | hsa-miR-665 | PHF1 | PHD finger protein 1 |
| 808 | 53 | hsa-miR-665 | IKBKE | inhibitor of nuclear factor kappa B kinase subunit epsilon |
| 827 | 52 | hsa-miR-665 | MEX3A | mex-3 RNA binding family member A |
| 836 | 52 | hsa-miR-665 | MTFR1L | mitochondrial fission regulator 1 like |
| 849 | 52 | hsa-miR-665 | ASB11 | ankyrin repeat and SOCS box containing 11 |
| 852 | 51 | hsa-miR-665 | LSP1 | lymphocyte specific protein 1 |
| 860 | 51 | hsa-miR-665 | DLG5 | discs large MAGUK scaffold protein 5 |
| 863 | 51 | hsa-miR-665 | KHNYN | KH and NYN domain containing |
| 865 | 51 | hsa-miR-665 | C1orf21 | chromosome 1 open reading frame 21 |
| 871 | 51 | hsa-miR-665 | JPH4 | junctophilin 4 |
| 877 | 51 | hsa-miR-665 | MLEC | malectin |
| 879 | 51 | hsa-miR-665 | SRCIN1 | SRC kinase signaling inhibitor 1 |
| 883 | 51 | hsa-miR-665 | TET3 | tet methylcytosine dioxygenase 3 |
| 887 | 51 | hsa-miR-665 | PHC2 | polyhomeotic homolog 2 |
| 889 | 51 | hsa-miR-665 | ZNF10 | zinc finger protein 10 |
| 899 | 50 | hsa-miR-665 | PDPK1 | 3-phosphoinositide dependent protein kinase 1 |
| 911 | 50 | hsa-miR-665 | PLAGL2 | PLAG1 like zinc finger 2 |
